# Investigation on Erosion Resistance in Polyester–Jute Composites with Red Mud Particulate: Impact of Fibre Treatment and Particulate Addition

**DOI:** 10.3390/polym16192793

**Published:** 2024-10-01

**Authors:** Sundarakannan Rajendran, Vigneshwaran Shanmugam, Geetha Palani, Uthayakumar Marimuthu, Arumugaprabu Veerasimman, Kinga Korniejenko, Inna Oliinyk, Herri Trilaksana, Vickram Sundaram

**Affiliations:** 1 Department of Mechanical Engineering, Saveetha School of Engineering, Saveetha Institute of Medical and Technical Sciences, Chennai 602105, India; sundarakannan.r@gmail.com (S.R.); s.vigneshwaren@gmail.com (V.S.); kesangee@gmail.com (G.P.); 2 Department of Mechanical Engineering, Center for Flexible Electronics & Advanced Materials, Amrita Vishwa Vidyapeetham, Amritapuri, Kollam 690525, India; uthay@am.amrita.edu; 3 Faculty of Mechanical Engineering, Kalasalingam Academy of Research and Education, Krishnankoil 626126, India; v.arumugaprabu@klu.ac.in; 4 Faculty of Materials Engineering and Physics, Cracow University of Technology, Al. Jana Pawła II 37, 31-864 Kraków, Poland; 5 Department of Materials Science and Engineering, Pryazovsky State Technical University, 87500 Mariupol, Ukraine; oleynik_i_m@pstu.edu; 6 Department of Physics, Faculty of Science and Technology, Airlangga University, Surabaya 60115, Indonesia; 7 Department of Biotechnology, Saveetha School of Engineering, Saveetha Institute of Medical and Technical Sciences, Chennai 600077, India; vickramas.sse@saveetha.com

**Keywords:** polymer composites, fibre treatment, jute fibre, erosive wear, sustainable development

## Abstract

This research investigates the manufacturing and characterisation of polyester-based composites reinforced with jute fibres and red mud particulates. The motivation stems from the need for sustainable, high-performance materials for applications in industries, like aerospace and automotive, where resistance to erosion is critical. Jute, a renewable fibre, combined with red mud, an industrial byproduct, offers an eco-friendly alternative to conventional composites. The composites were fabricated using compression moulding with varying red mud contents (10, 20, and 30 wt.%) and a fixed 40 wt.% of jute fibre. Fibre treatments included sodium hydroxide (NaOH) and silane treatments to improve bonding and performance. Erosion tests were performed using an air-jet erosion tester, examining the effects of the red mud content, fibre treatment, and impact angles. Scanning Electron Microscope (SEM) analysis provided insights into the erosion mechanisms. A distinctive reduction in erosion rates at higher impact angles (30°–60°) was observed, attributed to the semi-ductile nature of the composites. The addition of red mud enhanced erosion resistance, although an excess of 30 wt.% reduced resistance due to poor surface bonding. Silane-treated composites showed the lowest erosion rates. This study provides new insights into the interplay among material composition, fibre treatment, and erosion dynamics, contributing to the development of optimised, eco-friendly composite materials.

## 1. Introduction

Solid particle erosion in materials is a common problem in abrasive environments, particularly in natural fibre composites, where it causes significant damage and failure. Surface degradation, material loss, and compromised structural integrity are all mechanical consequences of abrasive wear in composites [1,2]. The continuous impact of abrasive particles has negative consequences, including the formation of microcracks, delamination, and changes in surface topography [3]. Cumulative damage significantly reduces the mechanical properties and overall performance of the composite material [4,5]. As a result, the erosion resistance of natural fibre composites must be improved. Fillers are emerging as a strategic intervention to mitigate the erosive impact [6,7,8]. The addition of fillers to natural fibre composites improves their resistance to abrasive wear, reducing erosion-induced material loss [9,10]. The filler particles act as sacrificial elements, absorbing and dispersing the kinetic energy of abrasive particles impinging on them. This method reduces the formation of microcracks and delamination, as well as the negative changes in surface topography [11]. As a result, the structural integrity of the composite is preserved, and the mechanical properties of the composites are better maintained in abrasive environments.

Red mud, also known as bauxite residue, is a byproduct of the extraction of alumina from bauxite ore. It is a highly alkaline waste material with significant environmental and economic consequences [12,13]. The global production of red mud is significant, with an estimated 150 million tons produced each year. The elevated pH levels of red mud pose environmental concerns, particularly when improperly disposed of, as it can contaminate soil and water [14]. Aside from its environmental challenges, red mud has sparked interest due to its potential use in a variety of applications. Efforts are being made to turn red mud into a valuable resource by researching applications in construction materials, geopolymers, and composite materials [15,16,17]. The challenges and opportunities in red mud disposal highlight the importance of sustainable waste management practices and innovative approaches to harness its potential for beneficial applications. Due to its particulate nature, red mud can be used as a reinforcing filler in composite fabrication [18,19]. When red mud is incorporated into polymer matrices, it has the potential to improve the mechanical properties of composites. Its inclusion can contribute to increased strength and stiffness, providing a long-term solution for improving the composite material performance [16,20]. Previous research has shown that the addition of red mud improves composite performance, specifically the surface hardness of the composites [18,21]. The increased hardness may benefit the composite by enhancing solid particle erosion. The composite erosion behaviour, which is influenced by the interaction between the matrix and reinforcing red mud particles, becomes an important factor in optimising materials for increased durability [11,22,23]. This incorporation of red mud into composite materials not only addresses environmental concerns associated with red mud disposal but also unlocks a sustainable solution for improving erosion resistance in a variety of industrial settings.

Enhancing the erosion resistance of natural fibre composites requires an improvement in fibre–matrix bonding. As mentioned earlier, numerous studies emphasise the role of fillers as agents that contribute to enhanced fibre–matrix bonding, thereby promoting improved interfacial adhesion and strengthening the structural integrity of composites [24,25]. Another widely employed technique in natural fibre composites is chemical treatment, considered the most reliable method for enhancing fibre–matrix bonding [26,27]. This involves modifying the surface chemistry of fibres to enhance compatibility with the matrix, thus fostering stronger bonds. It is anticipated that with improved interphase interactions, the composite becomes more resistant to erosive forces, reinforcing it against solid particle erosion in abrasive environments. Consequently, in the current study, both of these strategies were employed to enhance the erosion wear resistance of the composite. However, various treatment methods are utilised for natural fibre treatment [28,29,30], and it was found that sodium hydroxide (NaOH) and silane treatments were effective and cost-efficient. These two methods have been employed in this research.

In this study, hybrid composites were manufactured using polyester, jute fibre, and red mud. The weight percentage of jute fibre was kept constant at 40 wt.% based on the literature and the findings from our previous work, which indicates that 40 wt.% of natural fibre is optimal. The fibres were treated with NaOH and silane. The weight percentage of red mud was varied at 10, 20, and 30 wt.%. Composite fabrication was carried out using the compression moulding process, and subsequently, an erosion wear test was conducted at various impact angles. The insights of this investigation are instrumental for the ongoing efforts to refine and optimise composite materials for diverse applications, ranging from automotive to aerospace industries, paving the way for further exploration and refinement in the field of hybrid composites and offering a comprehensive understanding of their behaviour under erosive conditions.

## 2. Materials and Methods

### 2.1. Materials and Samples Preparation

The matrix material used was unsaturated isophthalic polyester resin, specifically Grade VBR 2303, which was obtained from Vasavibala Resins Private Limited in Chennai, India. GVR Enterprise in Madurai, India, provided jute fibre in the form of a woven mat.

**Figure 1:**
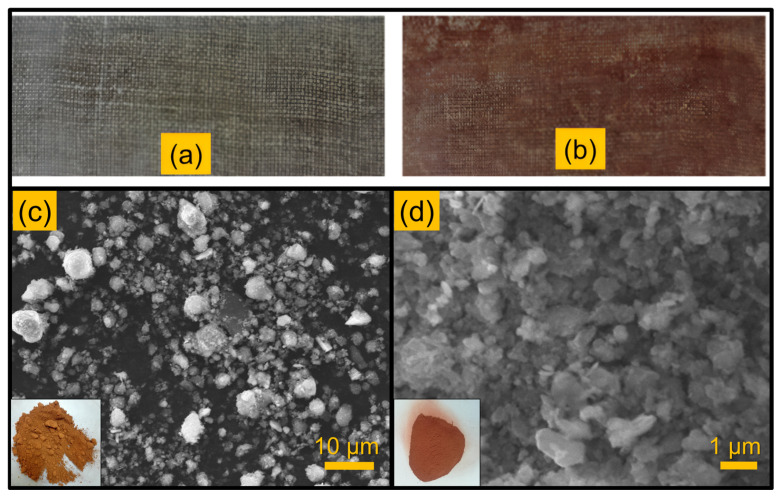
Fabricated laminate composites. (**a**) Unfilled jute laminate composite, (**b**) 10 wt.% red mud-filled jute laminate composite, (**c**) SEM image of red mud before sieving, and (**d**) after sieving.

The silane chemical was procured from Sigma Aldrich I.L.E.CO, Madurai, India. As particulate reinforcement, red mud was used, which was obtained from the National Aluminium Company Ltd.’s facilities in Damanjodi, India. The collected red mud was subjected to a ball milling process to ensure uniformity. Figure 1c,d depicts the morphological differences between sieved and sieved red mud. The average particle size after sieving was 0.7 µm.

The SANTEC Compression moulding supplied by Gurugram, Haryana, India was used in the laminate composite fabrication process. A steel mould and matching die were utilised having the dimensions of 300 × 135 × 3. The fibres were cut according to the size of the mould and were placed in the mould cavity. The fibre weight percentage was maintained constant at 40 wt.%. The matrix solution was prepared with varying percentages of red mud, such as 10, 20, and 30 wt.%. The required amount if matrix solution was taken and the red mud was added to it. The mixture was thoroughly stirred for about 15 min to ensure that the red mud was evenly distributed throughout the matrix. Following proper mixing, the catalyst methyl ethyl ketone peroxide and the accelerator cobalt naphthenate, each at a 1% concentration, were incorporated into the blend, as per the supplier recommendations. The mixture was stirred again. Following that, the resin mixture was poured onto the fibre layers in the mould cavity and rolled with a roller to remove any air bubbles. The mould was then sealed with the corresponding die and compressed at 150 kg/cm^2^ pressure. The laminate composite was allowed to cure at room temperature for 4 h. To facilitate the removal of the fabricated laminate composite plate, both the mould and matching die were applied with a releasing agent wax. Composites without red mud were also made using a similar process for comparison. In Figure 1a,b, fabricated laminate composites are shown.

Details of the fabricated laminate composites are shown in Table 1.

The fibres were alkaline treated (AT) with sodium hydroxide (NaOH) to remove the outer coarse layer and other natural contaminants from the fibre surface. The alkaline solution was prepared with the distilled water and 5% NaOH. The fibres were immersed in the prepared solution and treated for 3 h. Following that, the fibres were cleaned to remove any NaOH particles that had adhered to the fibre surface. Then, the fibres were dried to remove any residual moisture content. A similar process was used for silane treatment in a parallel approach. A 2% Triethoxy(ethyl)silane concentration was used to create the silane solution. For silane treatment, the NaOH-treated fibres were used (mentioned as AST).

### 2.2. Characterisation Methods

#### 2.2.1. X-ray Diffraction

The X-ray diffraction (XRD) patterns for both treated and untreated fibres were generated using the Bruker D8 Advance ECO X-ray diffractometer (Brucker, Billerica, MA, USA), which utilises a combined two-theta/theta scanning technique. These patterns were recorded at room temperature (25 °C) with a step size of 0.02. To ensure accurate diffraction patterns, a copper (Cu) anode was employed for the powdered samples. The Crystallinity Index (CI) of the fibres was determined using the Segal empirical method, as specified by Equation (1).

(1) $$\mathrm{CI}=\frac{I_{002}-I_{\mathrm{am}}}{I_{002}}\times 100,$$

where I_002_ is the maximum intensity of the crystalline peak and I_am_ is the minimum intensity of the amorphous material calculated from the XRD pattern.

#### 2.2.2. Fourier Transform Infrared Spectroscopy

Fourier Transform Infrared Spectroscopy (FTIR) was employed for the characterisation of both treated and untreated fibres, utilising the SHIMADZU IR Tracer 100 FTIR spectrophotometer (Shimadzu, Duisburg, Germany). The KBr Pellets technique was used, and the fibres are finely ground and mixed with potassium bromide (KBr) powder.

#### 2.2.3. Erosion Test

**Figure 2:**
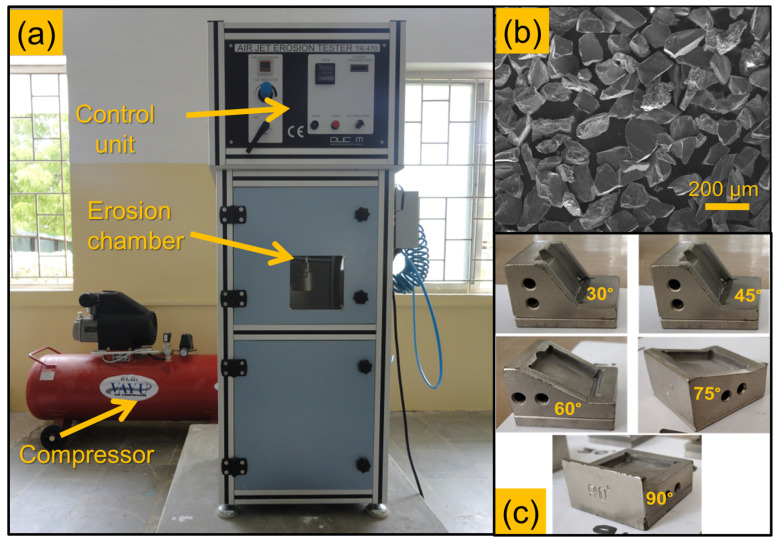
Erosion investigation: (**a**) erosion test rig used, (**b**) morphology of different erodents, and (**c**) different sample holders used.

The erosion wear behaviour of the composites was evaluated using the Air-jet erosion tester TR-470, supplied by Ducom Instruments in Bangalore, India. Samples with dimensions of 25 × 25 × 3 mm were employed for the erosion tests. Figure 2a depicts the erosion test rig utilised in the study. The setup includes a compressor unit responsible for supplying dry compressed air, an erodent feeder unit, and an erosion chamber. Within the erosion tester, the erodent from the feeder unit is carefully blended with dry compressed air and subsequently directed through a tungsten carbide nozzle, which measures 50 mm in length and 1.5 mm in diameter. During the test, the work sample was securely positioned in the sample holder, maintaining a nozzle tip-to-sample surface distance of 10 mm. For the erosion tests in this investigation, aluminium oxide with an average particle size of 50 microns was used as the erodent. The morphology of the erodent particles is visualised in Figure 2b. This experimental setup allowed for the systematic assessment of the erosion wear resistance of the laminate composite materials under controlled conditions.

During the erosion examination, high-abrasion particles expelled from the nozzle impacted the specimen, leading to material loss. The erosion trials lasted for 10 min, with the impact angle varying between 30°, 45°, 60°, 75°, and 90°, while maintaining a constant velocity of the erosive agent at 100 m/s. The holder, which can be adjusted to accommodate different impact angles, is illustrated in Figure 2c. To determine the velocity of the abrasive particles striking the specimen, the double-disc method was utilised. The mass flow rate of the abrasive particles remained constant at 3.3 g/min. Given that the primary concern is the loss of mass, the weight of each sample was recorded before and after the erosion trial. To ensure accuracy, each sample underwent three trials, and the average weight loss was calculated. Using the weight loss data, the erosion rate (Er) for each sample was determined using Equation (2).

(2) $$E_{r}=\left(W_{\mathrm{before}}-W_{\mathrm{after}}\right)/\left(T\times D\right),$$

where

*W*_before_ = weight of specimen before testing,

*W*_after_ = weight of specimen after testing,

*T* = time taken for erosion testing,

*D* = erodent discharge rate.

#### 2.2.4. Scanning Electron Microscope (SEM)

The morphology, microstructural characterisation, and failure analysis of the fabricated composites were analysed using the Scanning Electron Microscope (SEM)—ZEISS EVO 18 (Jena, Germany). Images were captured in secondary electron mode as per the specific analysis requirements.

## 3. Results and Discussion

### 3.1. FTIR and XRD Analysis

**Figure 3:**
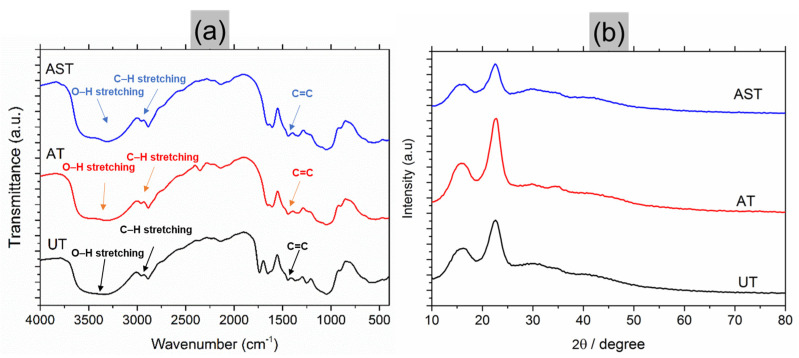
Specimen investigation: (**a**) FTIR result, (**b**) XRD result.

In Figure 3a, the FTIR spectra of untreated jute fibre, as well as those treated with NaOH and silane, are depicted. In the untreated fibres, the presence of lignin and hemicellulose structures in their composition was confirmed by the vibrational peaks observed at 1200 cm^−1^ and 1500 cm^−1^, respectively.

The presence of water absorption was indicated by a peak at 1600 cm^−1^. Peaks at 2900 cm^−1^ indicated the presence of cellulose and hemicellulose groups. The broad band observed in the range of 3000 cm^−1^ to 3700 cm^−1^ resulted from the hydrogen-bonded -OH vibration of the cellulosic structure. The wave numbers and their corresponding functional groups are provided in Table 2.

The analysis of vibrational peaks provides insight into the chemical modifications of fibres and their effects on composite properties. Peaks at 1200 cm^−1^ and 1650 cm^−1^ are indicative of specific functional groups present in the fibres. The peak at 1200 cm^−1^ is associated with C-O stretching vibrations, while the peak at 1650 cm^−1^ is attributed to C=C stretching vibrations. Reduced intensity at 1427 cm^−1^ suggests changes in the molecular environment and composition of the fibres, pointing towards a decrease in the presence of certain functional groups, likely associated with lignin. The absence of hemicellulose content in the fibres post-alkali treatment is a significant finding. Hemicellulose removal could lead to increased fibre crystallinity and improved mechanical properties due to the elimination of amorphous regions [31]. This increased crystallinity can result in fibres that are stiffer and stronger, contributing to a composite material with enhanced mechanical performance [32]. The presence of a vibration peak at 847 cm^−1^ indicates the formation of Si-C stretching bonds, confirming the presence of the silane component in the treated fibres. Silane treatments are often used to enhance the compatibility between fibres and polymer matrices in composite materials, improving adhesion and overall performance [33]. By forming a chemical bridge between the fibre and the polymer matrix, silane enhances interfacial bonding, which is crucial for effective load transfer and overall composite strength [34].

Broad peaks in the 1200 cm^−1^ to 1500 cm^−1^ range indicate the presence of lignin and hemicellulose structures in untreated fibres. The broadness of these peaks suggests a degree of structural heterogeneity and disorder in these components. The decrease in peak intensities, particularly following alkali treatment, signifies the removal and modification of lignin and hemicellulose components [35]. This could lead to improved fibre–polymer interactions and overall composite performance by creating a more uniform and cohesive interface [36]. The peak at 1734 cm^−1^ corresponds to the stretching of the C=O vibration in hemicellulose. Its decrease indicates hemicellulose removal or modification, corroborating the complete elimination observed in alkali-treated fibres.

The analysis leads to the conclusion that NaOH treatment removes cellulosic materials, such as hemicellulose, the wax content, and lignin. While the reduction of these cellulosic materials may impact the fibre’s strength, it enhances the compatibility of these fibres with the polymer matrix [37,38,39]. The presence of silane on the fibre surface contributes to increased interfacial bonding between the fibre and matrix, resulting in composites with superior mechanical properties and overall performance [34,40].

Figure 3b displays the XRD pattern of the fibre, revealing two peaks in all three patterns, consistent with defined XRD peaks for natural fibres reported by earlier research [24,41,42,43]. These peaks correspond to the cellulose I and IV components in natural fibres. Chemical treatment has a notable effect on the crystalline nature of the fibre.

Table 3 presents the crystallinity index values derived from the XRD analysis.

Treatment with NaOH noticeably enhances the crystallinity of the fibre in comparison to the untreated counterpart. This enhancement stems from the interaction between NaOH and the cellulose within the fibre, resulting in the elimination of non-cellulosic components. Consequently, this process fosters tighter packing of the cellulose chains, thus elevating crystallinity. The improved packing contributes to enhanced bonding with the matrix and subsequently augments the mechanical strength. The silane-treated fibre demonstrates a contrasting trend. Although its crystallinity is lower than that of NaOH-treated fibres, it surpasses that of the untreated fibre. The interaction between silane and cellulose on the fibre surface induces the formation of hydrogen bonds with cellulose chains, transitioning the surface crystallinity from crystalline to amorphous. The subsequent diffusion of the silane coupling agent through the developed amorphous region on the surface initiates a reaction with the crystalline cellulose, resulting in the generation of additional amorphous cellulose.

### 3.2. Solid Particle Erosion Test

**Figure 4:**
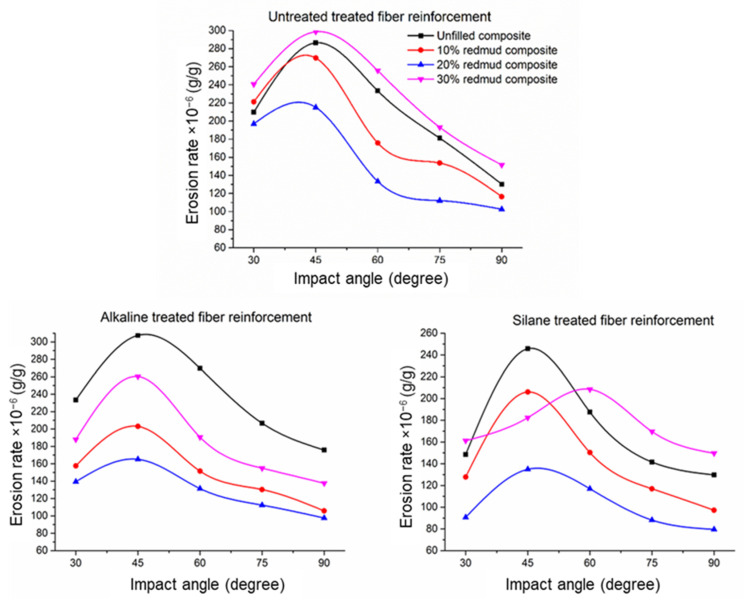
Erosion test results of red mud jute laminate composites.

The assessment of solid particle erosion is notably impacted by both the parameters of the erosion test and the properties of materials, especially within the realm of polymer composites. Fundamental erosion test parameters, such as the impingement angle, erodent velocity, and erodent flow rate, are pivotal in determining the erosion rate of polymer composites. This erosion rate within polymer composites typically fluctuates, largely dependent on the specific type of fibre and filler reinforcement utilised. Figure 4 visually depicts the fluctuation in erosion rates of these composites across different impact angles. It is observed that the erosion rate varies concerning both the composite material and the angle of impact.

#### 3.2.1. Erosion Rate at Different Impact Angles

In the context of polymers, erosion behaviour is intricately linked to the impact angle of abrasive particles. In general, the range of 30° to 60° impact angles has been identified as the zone where polymers exhibit the maximum erosion [11,44]. Interestingly, a discernible trend emerges from the current experimental setup, revealing a reduction in the erosion rate as the impact angle increases. This trend is noteworthy as it deviates from conventional expectations for ductile and brittle materials. Typically, ductile materials showcase maximum erosion at impact angles between 15° to 30°, while for brittle materials, erosion peaks at a 90° impact angle [6,15]. However, in the present study, the maximum erosion rate consistently occurs within the range of 30° to 60° impact angles, regardless of the composite’s material composition [45,46].

At lower impact angles, abrasive particles tend to slide along the surface, leading to a combination of ductile and brittle failure modes. The sliding motion at these angles creates micro-cutting and ploughing mechanisms, resulting in both material removal through plastic deformation (ductile failure) and some crack initiation (brittle failure), depending on the toughness of the matrix and filler. As the impact angle increases, particularly in the range of 30°–60°, the material begins to exhibit predominantly plastic deformation. At these higher angles, the composite absorbs the impact energy more effectively, allowing the polymer matrix to deform plastically without significant cracking. This plastic flow helps in reducing wear as the material dissipates energy, and the strain hardening mechanism further enhances its resistance. The fillers, like red mud, reinforce the matrix and limit excessive deformation, improving the overall wear resistance. At even higher impact angles (closer to 90°), the material undergoes primarily plastic deformation with minimal brittle failure, as the normal stress component increases, allowing the composite to withstand the abrasive forces without significant fracturing. Thus, the semi-ductile nature of the composite is characterised by a transition from mixed failure modes at lower angles to predominantly ductile behaviour at higher angles, with plastic deformation playing a dominant role in reducing wear.

The unique observation points towards the semi-ductile nature exhibited by the composites under investigation. The semi-ductile behaviour observed in the laminate composites can be attributed to the reinforcing effects imparted by both fibres and particles present in the laminate composite material. These reinforcing elements alter the erosion dynamics, deviating from the expected behaviour in purely ductile or brittle materials. The fibres and particles act as effective energy absorbers, mitigating the impact force and altering the deformation mechanisms.

The complex interplay between material composition, reinforcement effects, and erosion behaviour becomes apparent in the observed characteristics. The presence of reinforcing elements not only enhances the hardness of the composites but also plays a pivotal role in modifying the response to erosive forces. This deviation from traditional erosion behaviour underscores the nuanced and multifaceted nature of erosion in composite materials, where the interplay between various components leads to distinctive material responses under abrasive conditions. The insights gained from these observations contribute to a deeper understanding of erosion mechanisms in polymer laminate composites and provide valuable information for optimising material design and performance.

#### 3.2.2. Effect of Red Mud and Fibre Reinforcement

Across all the laminate composites studied, it was observed that the maximum erosion rate was exhibited by unfilled and 30 wt.% red-mud-filled laminate composites, while the minimum erosion rate was noted in the 20 wt.% and 10 wt.% red mud laminate composites. This observation underscores the development of erosion resistance with the addition of red mud, thereby enhancing the overall durability of the laminate composites. The kinetic energy imparted by the erodent is efficiently absorbed by the red mud particles dispersed within the matrix. Consequently, these particles act as energy absorbers, mitigating the impact force on both the matrix and the fibre reinforcement. As a result, material removal is minimised, with deformation primarily occurring in the form of plastic deformation. This synergy contributes significantly to the laminate composite’s remarkable erosion resistance. Moreover, the even distribution of particles throughout the polyester matrix enhances the bonding strength. However, a notable observation arises when the red mud content is increased to 30 wt.%, leading to a decline in erosion resistance compared to laminate composites reinforced with 10 wt.% and 20 wt.% red mud. This decrease is attributed to the tendency of the filler to excessively accumulate on the matrix surface, weakening the bonding interface between the matrix and filler. Consequently, erosion resistance is compromised as the erodent can easily remove the surface layer, accelerating the attack on the fibres.

With the continuous impact from abrasive particles, fibres undergo bending and breakage. When exposed to erosive forces for matrix layer removal, erodent particles initiate an attack on the fibre surfaces. Due to the inherently poorer erosion-resistance properties of fibres, they become more susceptible to erosion, resulting in increased damage, such as fragmentation and fibre removal. The accelerated erosion rate is due to the removal of both fibres and the matrix, highlighting the significant influence of the material’s morphological structure on erosion dynamics. When hard-abrasive particles impact the material surface, their kinetic energy is converted into the impacted area, causing plastic deformation and separating the reinforcement from the matrix. Simultaneously, the laminate composite surface experiences damage, including micro-cuts and craters, due to repetitive impact from hard erodent particles. This dual mechanism underscores the intricate interplay among the material properties, morphological structure, and erosion dynamics in composite materials.

#### 3.2.3. Erosion Rate and Treated Fibre Influence

Fibre treatment significantly impacts the erosion rate of composites, with the most notable decrease observed in silane-treated fibre-reinforced laminate composites, resulting in a minimum erosion rate. This reduction is primarily attributed to the improved bonding between the fibre reinforcement and the matrix. However, the effectiveness of fibre treatment diminishes in 30 wt.% laminate composites, suggesting that the treatment’s influence may vary depending on the specific composition and concentration of the laminate composite.

Figure 4 highlights the importance of fibre treatment in polymer laminate composites. Untreated fibre-reinforced laminate composites show the highest erosion rate, followed by NaOH-treated fibre laminate composites, indicating the efficacy of fibre treatment in enhancing erosion-resistance properties. The lower erosion rate in treated fibre composites is attributed to the establishment of strong interfacial bonding between the fibres and the matrix. A comparison between NaOH-treated and silane-treated laminate composites reveals that silane-treated laminate composites exhibit the lowest erosion rate. This improvement in erosion resistance is mainly attributed to the enhanced bonding achieved through silane treatment, which enhances the compatibility between the fibre surface and the matrix, resulting in a stronger and more durable interface.

The NaOH treatment on fibres removes natural and artificial surface impurities, such as waxes, oils, pectins, and hemicelluloses, effectively cleaning the fibres. This treatment disrupts hydrogen bonds in the cellulose, causing the fibres to swell and increasing their reactivity. Additionally, it converts cellulose I (native cellulose) to cellulose II (mercerised cellulose), altering the crystalline structure of the fibres. NaOH treatment also forms reactive alkoxide groups (–O–) on the fibre surface, which are highly reactive and ready for further chemical modifications. Following this, silane treatment involves hydrolysing the alkoxy groups (OR’) to form silanol groups (Si-OH), which then condense to create a siloxane network (Si-O-Si). These silanol groups react with the hydroxyl groups on the fibre surface, forming covalent Si-O-C bonds that graft the silane molecules onto the fibres. This process creates a crosslinked network on the fibre surface, enhancing the fibres’ compatibility with polymer matrices. Additionally, the introduction of specific functional groups, such as amino, epoxy, methacryloxy, and vinyl, tailors the surface properties for improved bonding, hydrophobicity, and chemical reactivity. This helped in enhancing the erosion resistance of the treated fibre composites. The increased crystallinity resulting from these treatments significantly improved fibre–matrix bonding, thereby enhancing the wear resistance of the composite. The transformation of native cellulose into a more crystalline form strengthened the fibres, improving their erosion resistance. Silane treatment further reinforced this effect by chemically bonding the fibre surface to the polymer matrix, forming a strong interfacial bond. This robust interface reduced wear damage, allowing the composite to resist abrasive forces more effectively.

The results emphasise the pivotal role of fibre treatment in optimising the erosion-resistance properties in polymer laminate composites. By tailoring the surface characteristics and interfacial bonding through treatments like silane application, it is possible to significantly enhance the overall performance and durability of the laminate composite material, particularly under erosive conditions. These findings contribute to the ongoing efforts to refine and optimise the design and functionality of laminate composite materials for various applications.

### 3.3. Morphological Analysis

**Figure 5:**
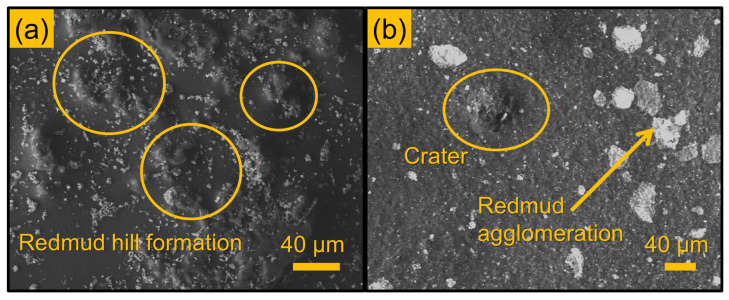
SEM observations: (**a**) crater development due to erodent impact, (**b**) red mud hill formation at 90° on 30 wt.% red mud—jute fibre laminate composite.

**Figure 6:**
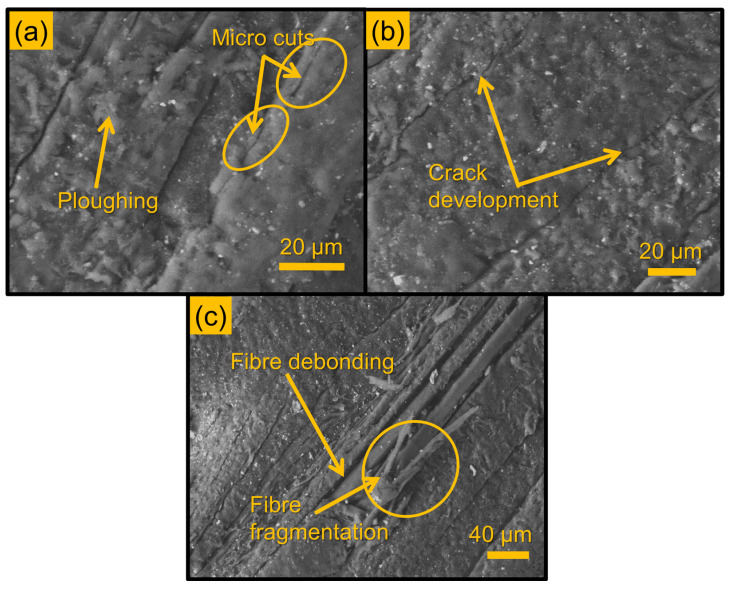
Crack, ploughing, and fibre damage due to erodent impact, (**a**) ploughing, (**b**) cracks, (**c**) fibre damage due to erodent impact.

**Figure 7:**
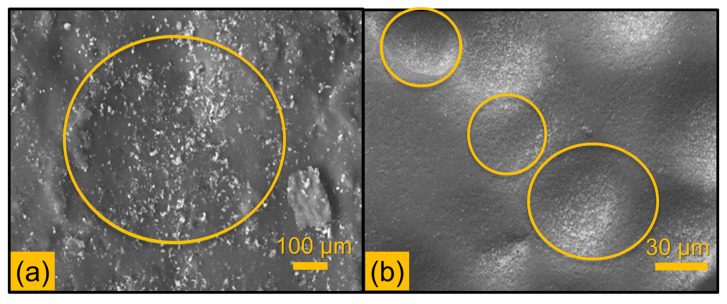
SEM images: (**a**) deep crater formation at 90°, (**b**) array of crater formation.

The erosion mechanism of the laminate composites was varied with respect to the impact angle, which was identified through the SEM images of the eroded surface (Figure 5, Figure 6 and Figure 7). The erodent’s impact readily removes the surface layer due to insufficient surface interface, accelerating the assault on the fibre. Figure 5a illustrates the buildup of red mud, while Figure 5b depicts the emergence of a hill-shaped formation on the laminate composite surface. Within this area, bonding is fragile, leading to the collapse of the hill structure upon erodent impact, resulting in the formation of micro-craters, cracks, and pores.

In both chemically treated laminate composites, the peak erosion rate is observed between impact angles of 30° and 60°. This variability is attributed to the bonding characteristics between the fibre and matrix. Despite experiencing maximum loss at different impact angles within the 30° to 60° range, all laminate composites demonstrate a semi-ductile behaviour, as the highest erosion occurs at lower impact angles.

At lower impact angles (30° and 45°), the surface displays extensive micro-cuts and severe ploughing (see Figure 6a), while at higher impact angles (90°), craters and cracks are evident (see Figure 6b). Greater material loss at lower angles is due to the sharp cutting edge of the erodent, which ploughs the laminate composite surface, resulting in surface material chipping off. With repeated impacts, the surface material is progressively removed, revealing the fibre surface (see Figure 6c). These exposed fibres are susceptible to damage and fragmentation from the impacting erodent particles (see Figure 6c). However, in the case of treated fibre reinforcement, the bond between the matrix and fibre mitigates surface damage from erosion.

For all the laminate composite materials, the lowest erosion rate was observed at a 90° impact angle, which can be attributed to the semi-ductile nature of the fibre laminate composite. At higher impact angles, particularly close to 90°, the normal force exerted by abrasive particles becomes dominant. This increase in normal stress leads to localised plastic deformation, causing the formation of craters as the material surface is directly impacted and displaced. The concentrated energy at the impact point induces severe stress, often exceeding the material’s yield strength, resulting in the creation of craters. Additionally, the repetitive nature of the impact initiates and propagates cracks. However, due to the semi-ductile behaviour of the material, the surface undergoes plastic deformation, reducing the likelihood of significant material loss. Continuous impact leads to cratering on the surface, as shown in Figure 7, but this plastic deformation, combined with strain hardening, helps limit wear. The accumulation of plastic strain and local tensile stresses results in the formation of cracks and craters at high impact angles, ultimately minimising wear loss.

Conversely, at lower impact angles, the erodent particles easily glide over the surface, causing micro-cuts and ploughing. Repeated impacts under this condition lead to more material removal from the surface, thus becoming the primary reason for the increased erosion rate at lower impact angles.

## 4. Conclusions

The study delved into examining the erosion characteristics of polyester laminate composites reinforced with red mud and sisal. The investigation unveiled a multifaceted interaction of variables that notably affect how the material reacts with abrasive forces. The impact angle of abrasive particles plays a pivotal role, highlighting the semi-ductile nature of the composites. This behaviour was attributed to the reinforcing effects of fibres and particles in the composite, acting as effective energy absorbers and altering erosion dynamics. The addition of red mud contributes to erosion resistance, with an optimal concentration observed at 20 wt%. Beyond this concentration (i.e., 30 wt.%), a decline in erosion resistance occurs due to excessive accumulation on the matrix surface, weakening the bonding interface. The morphological analysis reveals intricate mechanisms at play, with erodent impact causing micro-cuts, craters, and fibre damage. The importance of fibre treatment, particularly with silane, is underscored by the significant reduction in the erosion rate, emphasising the role of enhanced bonding between fibres and the matrix. The semi-ductile nature of the composites is further emphasised by the minimum erosion rate at a 90° impact angle. This behaviour is attributed to the absorption of impact energy by the matrix material, resulting in plastic deformation and craters on the composite surface. The findings highlight the nuanced relationship among the material composition, reinforcement effects, and erosion dynamics.

The results emphasise the complex link among the material composition, reinforcing effects, and erosion dynamics. Furthermore, the research shows that altering surface features and interfacial bonding via fibre treatment, such as silane application, can significantly improve erosion-resistance capabilities. These findings advance our understanding of erosion mechanisms in polymer composites and provide useful information for optimising material design and performance in a variety of applications.

## Figures and Tables

**Table 1:** Details of fabricated laminate composites.

Fibre	Fibre wt.%	Red Mud wt.%	Matrix wt.%
Untreated	40	0	60
	40	10	50
	40	20	40
	40	30	30
NaOH-treated	40	0	60
	40	10	50
	40	20	40
	40	30	30
Silane-treated	40	0	60
	40	10	50
	40	20	40
	40	30	30

**Table 2:** FTIR peak wavenumber and their functional group.

Bond Type	FTIR Peak Wave Number (cm^−1^)		
	UT	AT	AST
O–H stretching and hydrogen bonds	3338	3331	3335
C–H stretching	2943	2939	2943
C=O stretching of acetyl or carboxylic acid	1741	-	-
Absorbed H_2_O	1651	1658	1651
C=C	1427	1427	1427

**Table 3:** Crystallinity of jute fibre.

Treatment	Crystallinity (%)
Untreated sisal fibre	38.9
NaOH-treated sisal fibre	42.8
Silane-treated sisal fibre	42.4

## Data Availability

The original contributions presented in the study are included in the article, and further inquiries can be directed to the corresponding author.
